# Supplementary material for: Psycho-oncology practice for cancer patients during the pandemic lockdown in Italy: A qualitative mixed-method study with psychotherapists
Source: PLoS One. 2025 Apr 28;20(4):e0318241. doi: 10.1371/journal.pone.0318241 (PMC12036904; doi:10.1371/journal.pone.0318241)
Supplement: Attachment — Submitted filename: PONE-D-24-04536_LetterReview.docx [file pone.0318241.s002.docx]

Thank you for giving me the opportunity to review this manuscript about this pivoting time in psycho-oncology history. The article was well-written and multi-layered. There were many relevant aspects that had important potential in terms of exploring the role and importance of psychotherapy within hospital settings.

Within the abstract, I suggest you change “the final dataset included 102 valid questionnaires” to “the final dataset included 102 valid responses”.

The introduction is clear although may be unnecessarily lengthy. I suggest that lines 65 to 81 be shortened to a sentence or two as I don’t think it is relevant to your research question. In addition, on lines 101-104 you mention that your article’s overarching goal is to promote psycho-oncology program. It is unclear to me in what ways this article will achieve this. Although I agree that your article advocates for more recognition of psycho-oncology professionals within hospital settings, have you thought about the ways in which you will disseminate your findings to stakeholders and policy makers within these hospital settings? Implementation science is the next big thing; how will your research contribute to the implementation of psycho-oncology care? Finally, should you have space, it may be important to further introduce/explain/breakdown what CIT is.

The methods are generally clear and straightforward. However, as mentioned above, it may be relevant to provide further explanations as to your used of CIT. I suggest you also provide more information on your use of triangulation. It may be worth, for reproducibility, to provide more details on how this “framework” was used/built. Finally, it is unclear to me why you asked your participants about previous involvement in emergency psychology. How does that impact their answers to your questions? In what ways is this important to keep in mind? Finally, it may be important to clarify that this is an inpatient setting, right from the beginning, as the situation may be vastly different in outpatient settings.

The results are well outlined, and themes appear clear and distinct. I suggest making theme 1 more explicit in the ways it relates to PTs’ practice and how they adapted. It appears to me that it is more strongly related to patients’ experience rather than PTs’ and therefore does not add much to your research question. In addition, the disclosure from patients could be further explained. How is this different than existential dread when facing potential death?

Finally, the discussion really ties it all together. I appreciated the clearer distinction between your two bigger themes: innovation and lack of recognition. I believe both have great potential but are too tangled in your results. I suggest taking this distinction which you have mastered in the discussion and applying it to the rest of the article. In addition, most themes seem to be rooted in systemic issues and situational factors. Can you further discuss the recognition of psychotherapy in hospitals using a public health or systemic lens? What do those results mean, what changes are needed? You may not need to spend as much time on ICTs.

Overall, this article addresses some important issues within psycho-oncology and sheds some light on its in hospital settings. Thank you for your submission.
